# Optimizing the location of point-of-care infant HIV testing devices in Zimbabwe to maximize impact and value: A modeling analysis

**DOI:** 10.1371/journal.pone.0350921

**Published:** 2026-08-28

**Authors:** Carolina Vivas-Valencia, Karen A. Webb, Clare F. Flanagan, Kudakwashe Takarinda, Elif Coskun, Anesu Chimwaza, Caitlin M. Dugdale, Adam Chindore, Kenneth A. Freedberg, Angela Mushavi, Mohammad S. Jalali, Andrea L. Ciaranello

**Affiliations:** 1 The University of Texas at San Antonio, San Antonio, Texas, United States of America; 2 Center for Health Technology Assessment, Massachusetts General Hospital, Harvard Medical School, Boston, Massachusetts, United States of America; 3 Organization for Public Health Interventions and Development, Harare, Zimbabwe; 4 Medical Practice Evaluation Center, Massachusetts General Hospital, Boston, Massachusetts, United States of America; 5 Zimbabwe Ministry of Health and Child Care, National PMTCT Program, Harare, Zimbabwe; 6 Division of Infectious Diseases, Massachusetts General Hospital, Harvard Medical School, Boston, Massachusetts, United States of America; 7 Division of General Internal Medicine, Massachusetts General Hospital, Harvard Medical School, Boston, Massachusetts, United States of America; University of Zimbabwe, Faculty of Medicine and Health Sciences, ZIMBABWE

## Abstract

**Background:**

Early HIV diagnosis in infants is essential for timely antiretroviral therapy (ART) initiation, but requires virologic testing – often performed in centralized laboratories, which can delay results and treatment initiation. Point-of-care (POC) testing delivers same-day results, facilitating prompt diagnosis and treatment, but availability is limited in low-resource settings. We aimed to optimally locate POC machines to improve life expectancy (LE) and net health benefit (NHB) for infants born to people with HIV in Zimbabwe.

**Methods:**

We linked the validated CEPAC-Pediatric HIV microsimulation model, populated with detailed subnational programmatic data, with a location-optimization model for 122 clinics in Matabeleland South’s 7 districts. Simulating two testing strategies – conventional laboratory-based and POC machine-based – we projected: 30-day result-return, 30-day ART initiation for children with HIV (CWH), LE, HIV-related healthcare costs, and NHB. We maximized LE or NHB for infants tested at 6–10 weeks old.

**Findings:**

With 17 POC devices in current locations, a projected 43.8% of tested infants would receive HIV test results, with 41.6% of CWH starting ART within 30 days. Undiscounted LE would be 67.85 years (25.91 among CWH), with average discounted lifetime costs of $202/infant. Optimizing locations to maximize LE would retain 6 devices in current locations and move 11 to new sites, leading to projected 52.6% 30-day result-return, 50.0% 30-day ART initiation, and 67.88 years LE (27.04 among CWH), costing $211/infant. Maximizing NHB with a willingness-to-pay threshold ≥ $1,164 (66% of Zimbabwe’s GDP *per capita*) would render the same optimal locations as maximizing LE. Additional optimally-located machines would further improve LE. Ensuring one machine per district while maximizing LE would require one additional machine and would increase 30-day result-return (to 53.3%), ART initiation (to 50.6%), and LE (to 67.88 years, 27.11 for CWH), costing $212/infant.

**Interpretation:**

Data-driven optimization of POC machine location could improve HIV detection and increase life expectancy and net health benefit for infants undergoing HIV testing in Zimbabwe.

## Introduction

Early infant HIV diagnosis (EID) for infants born to people living with HIV (PLWH) is critical to facilitate early antiretroviral therapy (ART) initiation, which can reduce the very high mortality observed among untreated infants with HIV by 76% [1,2]. In Zimbabwe, HIV prevalence among pregnant people is high at 13%, with 53,000 pregnant women receiving ART in 2021 [3,4]. However, while 84.1% of infants born to PLWH in Zimbabwe receive a diagnostic test within 2 months of birth [4], this does not always result in timely ART start. When conventional laboratory testing is used for EID, result-return at 1 month and ART initiation at 2 months can be low, e.g., 20% and 43% in a retrospective cohort study of 8 countries [5].

Broader use of point-of-care (POC) devices – portable devices that can be located within health clinics and deliver same-day results – could potentially improve infant HIV testing accessibility, result-return, and ART initiation in resource-limited settings [5,6]. In contrast to centralized laboratory-based testing, POC machines offer quick turnaround times without requiring sophisticated laboratory infrastructure, specimen transport to central laboratories, or specialized staff to operate machines, thereby reducing attrition between testing and result-return and improving rates of early ART initiation [5]. However, POC machines are limited in supply, and purchasing additional machines may be costly [7]. In addition, POC machines may not always function even if available due to limited availability of electricity, consumables (reagents and test cartridges), machine maintenance, trained staff, and other resources [8].

Optimizing POC device location could expand treatment for infants and extend their survival in both the short and long term. Location-optimization models provide a framework to identify the most efficient resource placement: maximizing or minimizing specified outcomes while accounting for constraints. In previous work, we developed a location-optimization model and found that optimally locating available POC machines in healthcare facilities in Matabeleland South, Zimbabwe would improve infant HIV test result-return and 30-day ART initiation [9]. This work examined only 30-day outcomes, and did not include costs or longer-term outcomes such as life expectancy (LE). Building upon this foundation, we now expand this work to optimally locate POC devices in order to maximize infant LE and net health benefit (NHB), a composite measure of value gained from healthcare investment.

## Methods

### Overview

We expanded the previously developed location-optimization model and linked it to the validated Cost-effectiveness of Preventing AIDS Complications Pediatric (CEPAC-P) model [9,10]. Our objective was to determine the optimal location of both currently available and potential additional POC machines in healthcare facilities in Matabeleland South, the province in Zimbabwe with the highest adult HIV prevalence (17.6%) [11], to maximize either LE or NHB for infants undergoing HIV testing at 6–10 weeks of age. At each facility, we modeled two mutually exclusive EID strategies using nucleic acid testing: 1) use of conventional laboratory testing for all specimens (*LAB*), and 2) placement of a point-of-care machine (*POC*) [12]. For both strategies, we projected clinical outcomes and costs for all infants tested, as well as for the subset of children with HIV (CWH).

This work was approved by the Massachusetts General Brigham Institutional Review Board (IRB). Programmatic data were accessed for the purposes of this research on 1 February 2022. De-identified aggregate data were shared with researchers. As such, no formal consent procedures were required, consistent with the approved IRB protocol.

### Modeled testing strategies

Under the *LAB* strategy, we assumed that all infants underwent conventional laboratory testing. Under the *POC* strategy, we defined a functionality term based on program data from the HIV services implementing agency, the Organization for Public Health Interventions and Development (OPHID), regarding the proportion of samples that are able to be successfully processed within a given week. We assumed that all specimens would be processed by the POC machine on days when it was functioning until its daily capacity was met. For these samples, we assumed a 98% 30-day result-return for specimens tested with a functioning *POC* machine. Samples collected on days when a POC was not functioning and those collected after the device’s daily capacity had been met would be sent out to undergo conventional laboratory testing. For *LAB*, 30-day result-return varied by facility from 27% to 43%, based on programmatic data (Table 1) [5]. As a simplifying assumption applied to both POC and conventional laboratory-based testing, after diagnosis is delivered, we assumed 95% of infants would initiate ART within 30 days [5]. ART was initiated upon receipt of the first positive result, and stopped if confirmatory test result was negative. Under both strategies, positive test results were confirmed with POC polymerase chain reaction tests. We did not specifically model hub-and-spoke testing scenarios due to a lack of data regarding the allocation of current testing between hub-and-spoke sites; however, the aggregate program data used in our analysis do reflect the testing outcomes from the current hub-and-spoke model at the province level.

**Table 1:** Model input parameters for a location-optimization analysis of point-of-care devices in Matabeleland South, Zimbabwe.

Parameter		Base-case value		Source
**I. Input parameters used in CEPAC-Pediatric model**				
**Infant cohort characteristics**				
Initial CD4% at infection, mean (SD), %		45 (9.4)		[13]
Pediatric ART regimens				
1^st^-line		ABC/3TC + DTG		[14, 15]
2^nd^-line		ABC/3TC + LPV/r		
3^rd^-line		AZT/3TC + DRV/r + DTG		
Adult ART regimens				
1^st^-line		TDF/3TC/DTG		[14, 15]
2^nd^-line		TDF + FTC + LPV/r		
3^rd^-line		TLD + DRV/r + DTG		
Probability of initial virologic suppression,^†^ one-time %				
Pediatric 1^st^-line ART		89		[16]
Pediatric 2^nd^- and subsequent lines of ART		87		[17]
Adult 1^st^-line ART		90		[18, 19]
Adult 2^nd^- and subsequent lines of ART		83		[20]
Monthly probability of becoming LTFU, %		0.41		[21]
**EID cascade parameters**		***LAB***	***POC***	
Time to test result-return, months (SD)		2(1)	0 (0)	Assumption
Test sensitivity		0.994	0.969	[22–25]
Test specificity		1	0.996	
**Cost data, 2020 USD**				
Conventional laboratory-based EID test		26.06		[26]
POC EID test		33.07		[22]
**II. Parameters used in location-optimization model linked to CEPAC-P**				
Test positivity rate, %		District-specific^‡^ (1.0, 4.3, 3.3, 2.5, 2.8, 1.7, 2.0)		Routine data: National AIDS and TB Program (2019)
Demand, # of infants seeking testing, by month		Health facility-specific		
30-day result-return to caregiver (%)	*LAB* EID	District-specific^‡^ (27, 37, 37, 43, 35, 15, 30)		
	*POC* EID	98**^††^**		[5, 27]
Number of POC machines		17		Routine data: National AIDS and TB Program (2023)
ART initiation upon positive result-return, %		95		Assumption [5]
Functionality^§^ of POC machines, %		District-specific^‡^ (70, 80, 35, 35, 90, 35, 98)		Estimate based on site observations over 2019

†Initial virologic suppression defined as <1,000 c/mL at week 48 after ART start.

**^††^** 98% 30-day result-return applies only to samples that are successfully processed by a POC machine (i.e., the machine is functioning when the sample is presented for testing and the machine has not met its daily capacity).

‡Beitbridge, Bulilima, Gwanda, Insiza, Mangwe, Matobo, Umzingwane^§^ Functionality defined as the proportion of days when a POC machine is able to process testing samples.

ABC/3TC, abacavir/lamivudine, ART, antiretroviral therapy; AZT, azidothymidine; DRV/r, darunavir/ritonavir; DTG, dolutegravir; EID, early infant diagnosis; FTC, emtricitabine; LPV/r, lopinavir/ritonavir; LTFU, loss to follow-up; POC, point-of-care; SD, standard deviation; TDF, tenofovir disoproxil fumarate; TLD, tenofovir, lamivudine, and dolutegravir; USD, United States dollars.

### CEPAC-Pediatric model

The CEPAC-Pediatric model is an individual-level, state transition model of pediatric HIV disease that has been previously validated using UNAIDS data from 12 sub-Saharan African countries and trial data from the IMPAACT P1060 trial based in multiple countries; both of these data sources included participants from Zimbabwe [13,28,29]. For this analysis, infants enter the model at birth and are simulated until death. At birth, modeled infants either: (1) have acquired HIV before/during delivery (CWH), or (2) have not acquired HIV. All infants are born to PLWH and are therefore considered high priority for EID. Additional details of CEPAC-P model structure are available in the Appendix and at: https://mpec.massgeneral.org/cepac-model/.

We used the CEPAC-Pediatric model to project LE and lifetime costs for three groups of infants born to PLWH and undergoing testing under both *POC* and *LAB* strategies: 1) children without HIV, 2) CWH who receive their results, and 3) CWH who do not receive their test results.

### Location-optimization model description and CEPAC-P linkage

We formulated an integer programming-based location-optimization model tailored to locate a user-specified number of POC machines at a specified number of healthcare facilities. The model was written in Python 3.8.6.2020 utilizing the Gurobipy function [30]. Facility‑level clinical and economic values under *POC* and *LAB* are pre‑computed for all 122 clinics, forming fixed‑coefficient vectors that feed directly into the 0–1 linear optimization model.

For each facility, the location-optimization model calculates LE and NHB under both the *POC* and *LAB* strategies by combining the sub-cohorts simulated in CEPAC-P. NHB is a measure that captures the overall impact of a healthcare intervention by balancing the health outcomes gained against the associated costs. NHB can be useful in aiding decision-making for efficient resource allocation [31].


$$\begin{array}{c} NHB=Discounted\;LE\;-\;\frac{Average\;discounted\;per-person\;life\;time\;cost}{Willingness-to-pay\;threshold} \end{array}$$ (1)

We computed NHB using LE and per‑person lifetime costs (both discounted at 3%/year) so that health and cost were evaluated on the same time‑adjusted basis within a single value expression. Discounting converts future streams to present value and, when applied consistently to both costs and health, avoids mixing time bases that would otherwise over‑weight long‑delayed benefits relative to present‑valued costs [32–34]. In the base case, we assumed a societal-level willingness-to-pay threshold (WTP) of $1,774/year-of-life saved (YLS) (Zimbabwe’s 2021 GDP *per capita*), and varied this from 0.5-2x GDP in scenario analyses, to reflect ongoing debate within the global health cost-effectiveness analysis field [35,36]. Although there is no consensus regarding the most appropriate cost-effectiveness threshold in resource-limited settings, the use of 0.5x per-capita GDP follows frequently recommended guidance which incorporates important opportunity cost tradeoffs when implementing new healthcare interventions [37,38].

Using the distribution of the six modeled sub-cohorts at each facility and their CEPAC-projected LE and lifetime costs, the location-optimization model calculates a total-cohort LE and NHB, assuming either placement or no placement of a POC machine in that site. The model then optimizes the location of a specified number of POC devices throughout the province, identifying the facilities at which POC machines should be placed to achieve one of two objective functions: maximal LE or maximal NHB. In the current scenario, actual placement reflected current location of more than one device in some facilities. In the optimized scenarios, we imposed a constraint to ensure that each clinic site was allocated either zero or one POC machine. Our analysis of programmatic data demonstrated that a single POC device was sufficient to meet the testing demand at each facility (Appendix).

### Model input parameters

We used program data from OPHID from 122 health facilities located in the seven districts of Matabeleland South from January 2019 to January 2020 (Table 1). In total, 4,408 infants underwent HIV testing using either POC or laboratory testing. From these program data, we derived facility-specific demand and district-specific test positivity rates, 30-day laboratory-based result-return, and POC machine functionality. For the modeled current scenario, 17 POC machines were available for EID and were located in 11 different health facilities, reflecting the actual placement of machines in Matabeleland South as of 2023. These 2023 locations were mapped onto 2019–2020 demand and programmatic data, which were the most recent available.

In the location-optimization model, the number of infants presenting for testing at each facility was informed by annual demand from Matabeleland South provincial data [39]. We used facility-specific test positivity rate as a proxy for true HIV prevalence among tested infants. See Appendix for details regarding data sources informing model inputs for HIV disease progression, CD4 and viral load trajectories, and ART use. CEPAC-P input derivation and calibration are described elsewhere [13,40].

Laboratory-based test costs came from the Global Fund [26]. We averaged the price for the Roche and Abbott platforms, using the cost at committed volumes (i.e., a reduced unit price for upfront purchases) for the total cost of ownership, which reflects logistics, equipment, set-up, servicing, training, controls, calibration, consumables, and reagent costs. POC test costs were derived from a Zimbabwe-specific analysis estimating the EID POC testing costs in programmatic settings; capital costs were amortized over the useful lifetime of the machines (5 years), added to costs for reagents, supplies, personnel time and training, electricity, maintenance, and other overheads, and divided by annual tests performed [22]. We averaged costs for four platforms. Original costs for both laboratory-based and POC tests were updated to 2020 USD using the GDP deflator (Table 1) [41].

### Model outcomes

The outcomes of the CEPAC-P model include discounted (3%/year) and undiscounted projected LE and HIV-related per-person lifetime costs, including both testing and HIV care and treatment costs, for the simulated infant sub-cohorts. (Note that while discounted LE is used to calculate NHB, in the Results, all reported LEs are undiscounted. All reported lifetime costs are discounted.) The outcome of the location-optimization model is the location assignment of each POC machine (based on the selected objective function, maximizing either LE or NHB) and the resulting LE, costs, and NHB for the overall cohort. Additional outcomes include the proportion of test results delivered to infants and caregivers within 30 days and the proportion of CWH initiating ART within 30 days of receiving a positive HIV test result.

### Sensitivity analyses

Following ISPOR-SMDM guidance for simulation model-based analyses [42,43], we conducted univariate and multivariate sensitivity analyses to assess the robustness of our optimal device allocation recommendations. We varied key clinical parameters in the CEPAC-Pediatrics model, including monthly risk of loss to follow-up from HIV care, risk of HIV transmission through breastfeeding to infants who did not acquire HIV in pregnancy/delivery, test performance characteristics, and key cost parameters (cost of ART, HIV care, and POC tests), individually and in combination across their plausible ranges. We did not conduct probabilistic sensitivity analyses as they are not appropriate for this linked modeling framework due to computational complexity and insufficient data on the probability distributions of influential parameters.

### Scenario analyses

*Adding point-of-care machines to the current program*. Relocating a POC device from an established location is likely to be logistically challenging and undesirable for healthcare workers and the populations they serve who are currently benefiting from POC testing. We therefore modeled a scenario in which the currently available 17 devices remained in their current location, and then we added new machines one by one, each time locating the new machine to maximize LE. There were no additional constraints placed upon the location of these new machines.

*Adding machines in a geographic equity scenario*. To explore the tradeoffs between pure efficiency and geographic fairness, we evaluated a scenario incorporating a geographic equity constraint. This constraint is minimal; it ensures one POC device per district but does not equalize access across population size or travel distance. This constraint serves as a pragmatic baseline policy goal. We began with the 17 currently available machines, located based on LE optimization. We then added one machine at a time, continuing to optimize LE while also ensuring that each district has at least one machine.

*Varying the willingness-to-pay threshold*. When maximizing NHB, we varied the WTP from $887/YLS to $3,548 (0.5-2x GDP *per capita*).

## Results

### Maximizing life expectancy

#### Base case.

**Fig 1:**
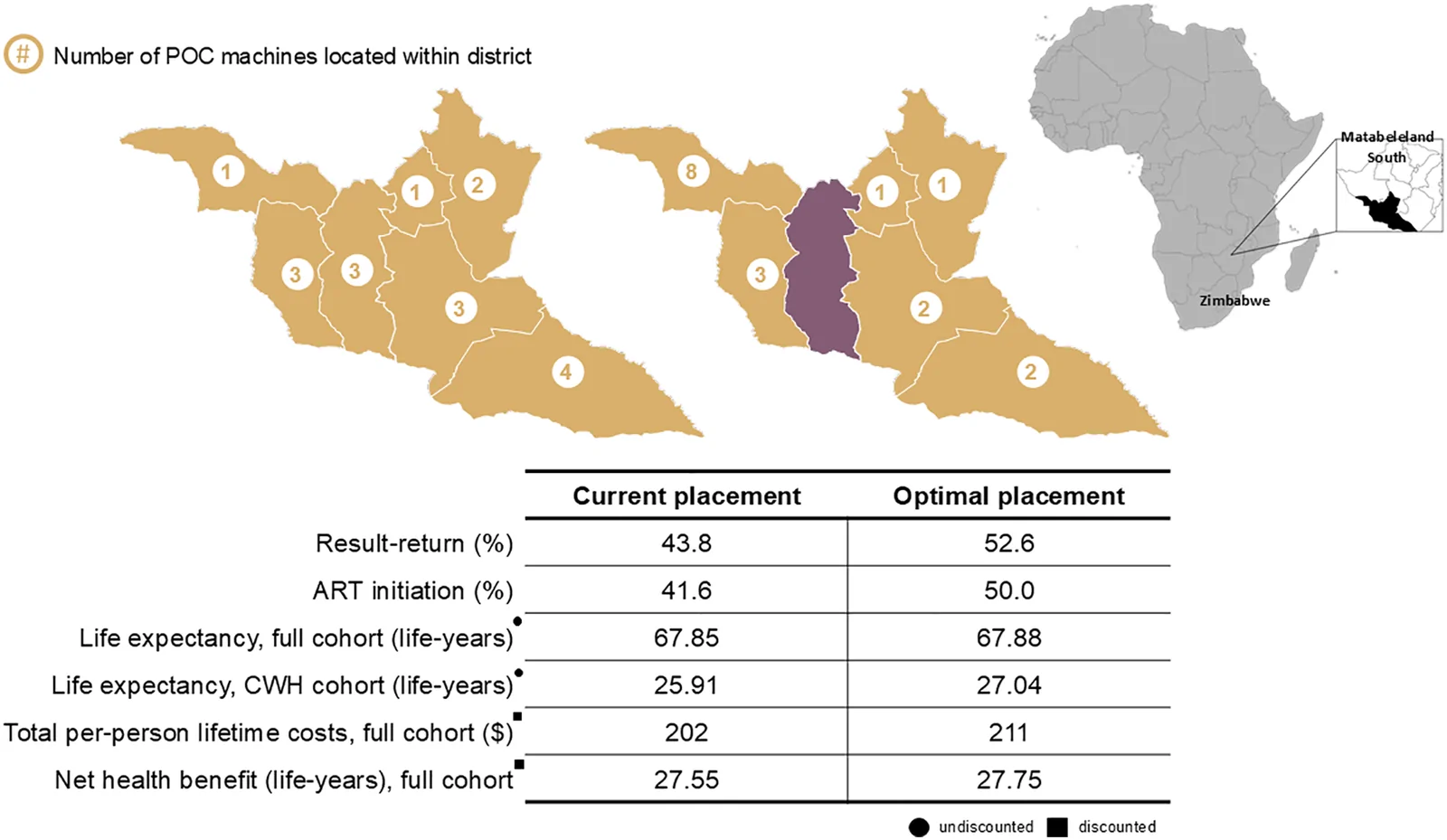
Current and optimal placement of 17 existing point-of-care machines, maximizing life expectancy. This figure shows the districts within the province of Matabeleland South. Current placement of the 17 available point-of-care (POC) machines shown is on the left and optimal placement, maximizing life expectancy (determined by a location-optimization model in conjunction with the CEPAC-Pediatric HIV microsimulation model), is shown on the right. Districts in brown indicate the location of POC machines, while districts colored in purple indicate the absence of a POC machine. Numbers inside each district represent the total number of POC machines located within that district. The table below displays corresponding projected health and economic outcomes. Health outcomes include % of all results returned, % of children with HIV (CWH) starting antiretroviral therapy (ART) and undiscounted life expectancy for the full cohort of infants undergoing HIV testing (including those with and without HIV), as well as for CWH. Economic outcomes include total discounted (3%/year) per-person HIV-related lifetime care costs and net health benefit (NHB), where NHB = discounted life expectancy – (average discounted per-person lifetime cost/willingness-to-pay threshold [WTP]). Here, WTP = $1,774/year-of-life saved, based on Zimbabwe’s GDP *per capita* in 2021. Under optimal placement (right), 6 machines would remain in the current location, and 11 would be moved to new sites.

With current placement of the 17 available POC devices, 43.8% of tested infants would receive results within 30 days, with 41.6% of CWH initiating ART within 30 days of testing (Fig 1). Projected undiscounted LE would be 67.85 years (25.91 for CWH), and average discounted lifetime cost would be $202/infant. Relocation of these 17 machines to maximize LE would leave 6 in their current location and move 11 to new sites. One district with low POC functionality (35% of days operational) and low test positivity rate (1.7% of tested infants) would not be allocated a POC device. Under this optimal location, 52.6% of tested infants would receive results within 30 days, 50.0% of CWH would initiate ART within 30 days of testing, undiscounted LE gains would be small at the population level (+0.03 life-years to 67.88 years, reflecting the small proportion of CWH within the full cohort), with larger gains accrued to CWH (+1.13 life-years, to 27.04 years). Discounted lifetime cost would be $211/infant.

#### Scenario analyses.

**Fig 2:**
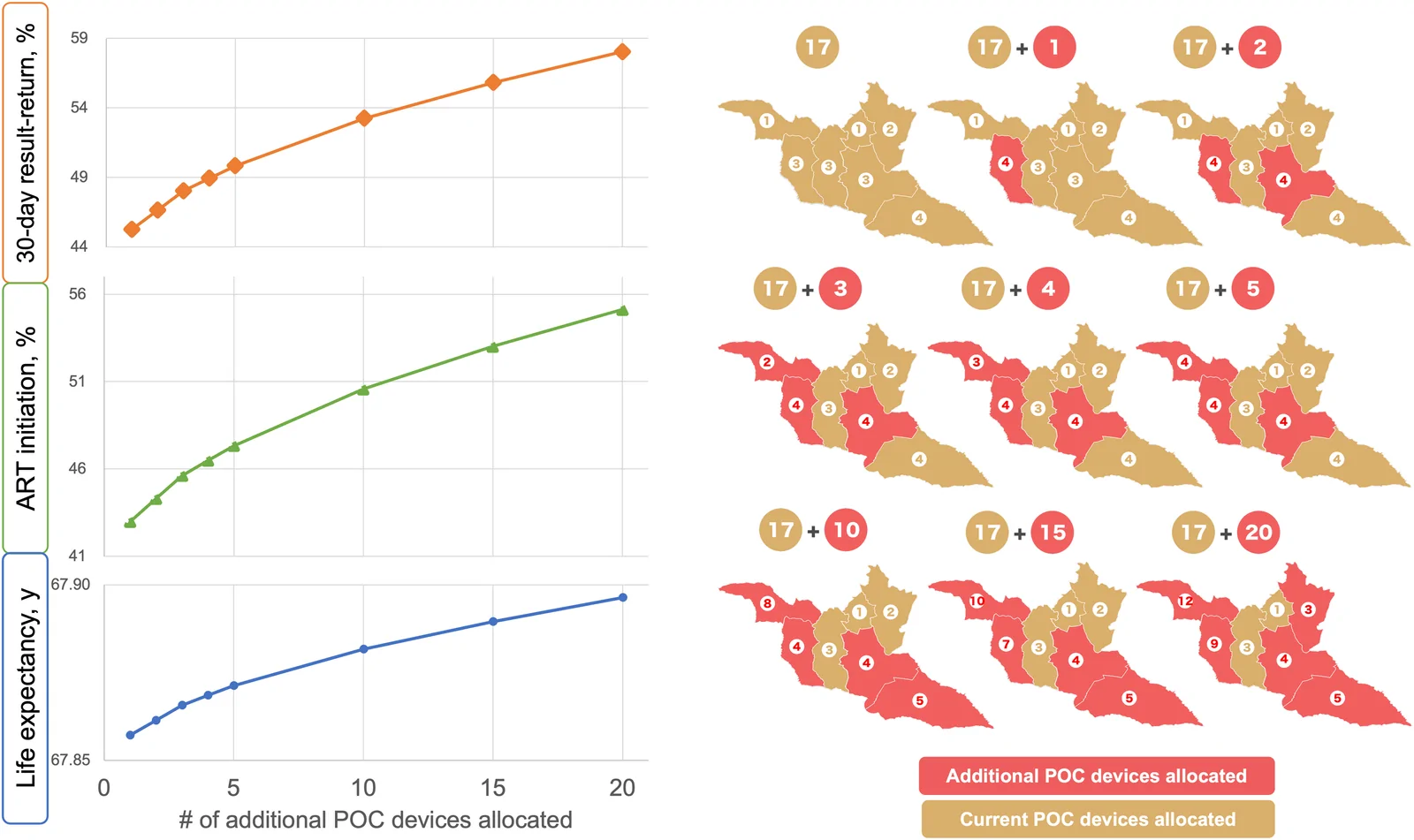
Scenario analysis: Adding additional machines to the 17 currently located POC machines, maximizing life expectancy. The maps represent the districts within Matabeleland South, Zimbabwe. Point-of-care (POC) device allocation is indicated for the current allocation (top row, left-most map) as well as additional hypothetical scenarios where currently available POC devices remain in their current facility, while additional POC devices become available and are allocated (maximizing life expectancy) by a location-optimization model, starting with 1 additional device (top row, middle graph), up to 20 devices (bottom row, right-most graph). Districts colored brown indicate the current locations of the 17 available point-of-care (POC) machines. When a district appears in red in a subsequent map, that indicates that that district has received one of the hypothetically newly available POC devices. The numbers within each circle on the map show the total number of POC machines available in that district. The left (brown) circle above a given map indicates the total number of currently available POC devices (17 throughout). The right (red) circle indicates the total number of newly available POC devices in that scenario. Districts with low POC functionality would not receive any new machines, even after 20 new machines were added. The increased number of POC machines would improve projected health outcomes, indicated by the graphs on the left-hand side of the figure, which show the health outcome on the y-axis and total additional number of available POC devices on the x-axis. Health outcomes include timely return of test results (top), 30-day antiretroviral therapy (ART) initiation (middle) and total cohort undiscounted life expectancy in years (bottom).

*Adding point-of-care machines to the current program*. Starting with the original placement of the 17 currently available machines and adding new machines one at a time with the goal of maximizing LE, the first 3 additional machines added would be located in districts with the highest test positivity rate. Even after adding 20 new machines, districts with low POC functionality were still not allocated a new POC device. As the number of POC machines increased, we observed increasing 30-day result-return, ART initiation, and LE (Fig 2).

**Fig 3:**
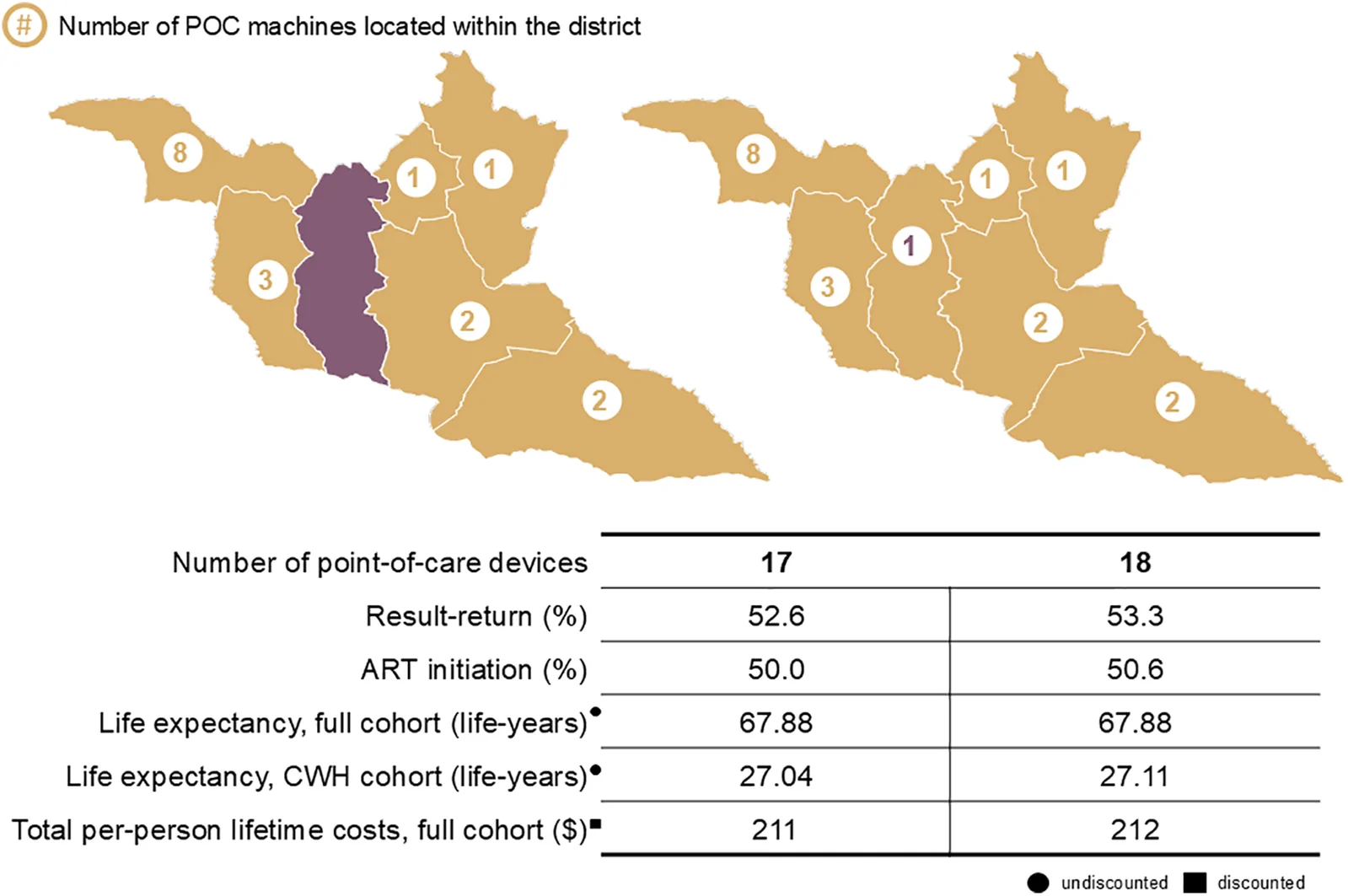
Optimal distribution of the 17 currently available POC machines and introduction of additional machines to ensure one per district, maximizing life expectancy. The figure shows a map of the districts within the province of Matabeleland South. On the left map, the optimal distribution of the currently available17 point-of-care (POC) machines maximizing life expectancy is depicted, as determined by a location-optimization model paired with the CEPAC-Pediatrics HIV microsimulation model. One district is colored in purple, indicating the absence of a machine. The right map depicts the optimal allocation of the 17 currently available devices plus one hypothetical additional device. All districts are colored in brown, signifying that each of the seven districts would be allocated a POC device if more were available. When adding more devices, only one additional POC device (bringing the total to 18) was identified as necessary to ensure both equitable and optimal distribution across districts. Health outcomes associated with each of the allocations are shown in the table below. These include % of all results returned, % of children with HIV (CWH) starting antiretroviral therapy (ART) and undiscounted life expectancy for the full cohort of infants undergoing HIV testing (including those with and without HIV), as well as for CWH. Economic outcomes include total discounted (3%/year) per-person HIV-related lifetime care costs and net health benefit (NHB), where NHB = discounted life expectancy – (average discounted per-person lifetime cost/willingness-to-pay threshold [WTP]). Here, WTP = $1,774 per year-of-life saved, Zimbabwe’s 2021 GDP *per capita*.

*Geographic equity scenario*. Starting with the base-case optimal placement of the 17 currently available machines and adding new machines one at a time to optimize LE, only one additional POC device was needed to ensure at least one machine per district. With these 18 machines, 53.3% of infants would receive results within 30 days, with 50.6% of CWH initiating ART within 30 days. Projected undiscounted LE would increase to 27.11 years for CWH and remain nearly unchanged (within rounding) at 67.88 years for all modeled infants. Under this scenario, discounted costs increased slightly, reaching $212/infant (Fig 3).

#### Sensitivity analyses.

With variation in key CEPAC-Pediatrics parameters such as monthly loss to HIV follow-up, breastfeeding HIV acquisition risks, and costs of ART, POC tests, and HIV-related care, we found anticipated differences in projected LE and lifetime costs (Table S4 in S1 Appendix). However, the optimal location of the 17 currently available machines when optimizing LE remained the same as in the base-case scenario.

### Maximizing net health benefit

#### Base case.

**Fig 4:**
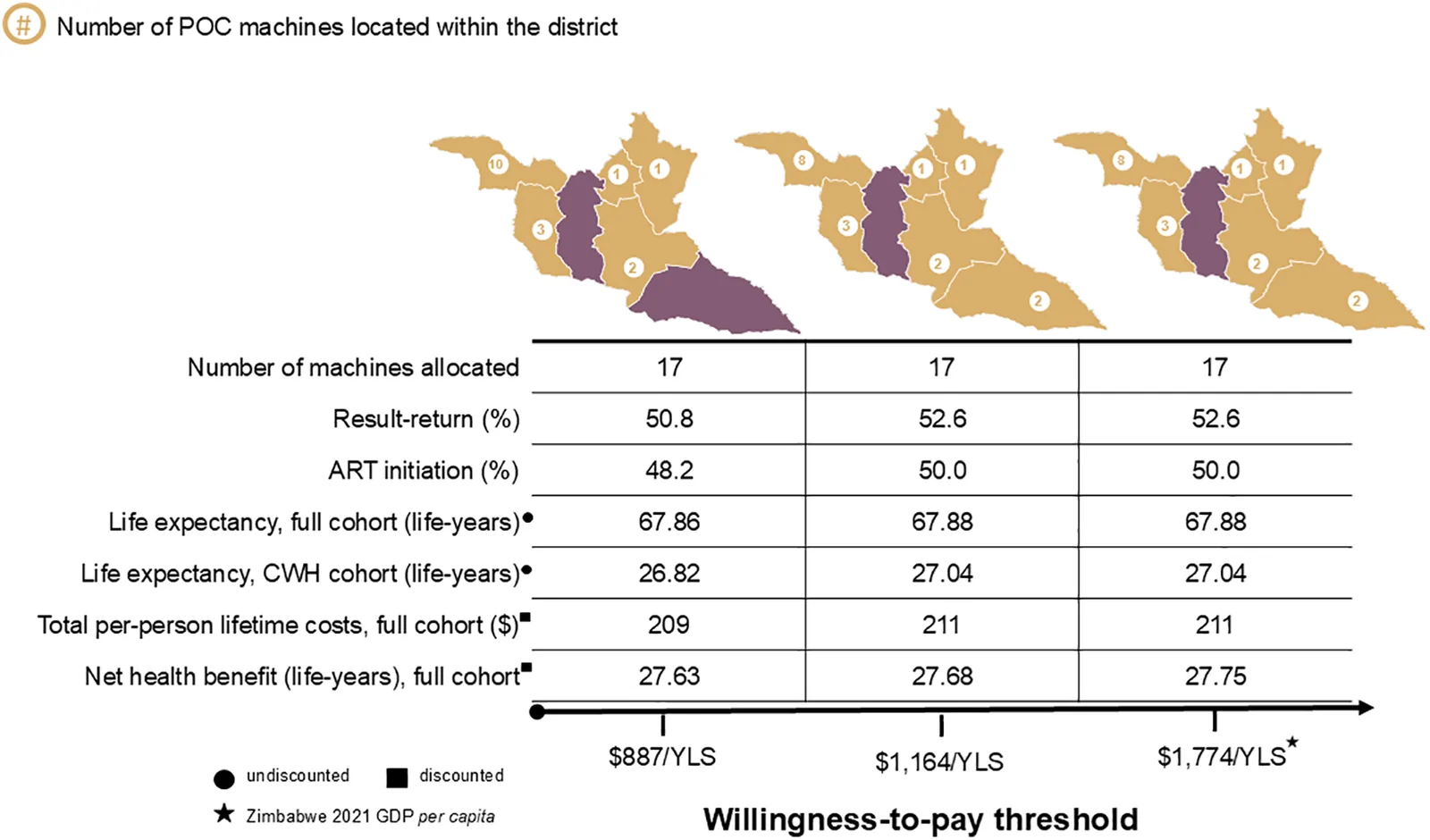
Optimal placement of POC machines, maximizing net health benefit at different willingness-to-pay thresholds. The impact of willingness-to-pay (WTP) thresholds on the placement of point-of-care (POC) machines and their effect on net health benefit (NHB) is illustrated in Figure 4. Each map displays the locations of POC machines within the districts of Matabeleland South, Zimbabwe at different WTP thresholds (increasing from $887 per year-of-life saved [YLS] on the left to $1,774/YLS on the right [0.5-1.0x Zimbabwe’s 2021 GDP *per capita*]). Districts shaded in purple indicate the absence of POC machines, while districts shaded in brown indicate the presence of POC machines and the number located therein. Variation in WTP leads POC machines to be allocated at different clinics, reflecting a balance of number of children tested at each clinic (impacting testing costs) and test positivity rate at each clinic (impacting number of children with HIV [CWH] who both gain life expectancy and accrue lifetime HIV care costs). As WTP increases, clinical outcomes for the modeled cohort improve, including projected result-return, 30-day antiretroviral therapy (ART) initiation and life expectancy, displayed in bottom table. At WTP values > $1,164/YLS, the optimal location of POC does not change with further increases in WTP, and calculated NHB increases without any change in projected life expectancy. Values greater than the base-case WTP of $1,774/YLS (1x GDP *per capita*) are not shown, as allocation of machines would not change.

When maximizing NHB utilizing a WTP of $1,774 (GDP *per capita*), optimal location of the 17 available POC devices led to the same solution and projected outcomes as the base-case solution maximizing LE: moving 11 devices, keeping 6 in current locations (Fig 4, right-most panel). In this scenario, 52.6% of tested infants would receive results within 30 days, 50.0% of CWH would initiate ART within 30 days, undiscounted LE would be 67.88 years (27.04 for CWH), and average discounted lifetime cost would be $211/infant.

#### Sensitivity analyses.

Varying monthly loss to follow-up, breastfeeding HIV acquisition risks and costs of ART, POC tests and HIV-related care led to changes in projected LE and lifetime costs and thus calculated NHB (Table S5 in S1 Appendix). Compared to the base case, the POC machine locations changed only with variation in POC testing costs. When POC test costs doubled, two machines located in facilities with low POC machine functionality and low test positivity rate were moved to facilities with higher functionality and positivity rate, and lower demand. When POC costs were halved, one machine was moved from a facility with lower demand to one with higher demand.

#### Scenario analyses.

*Varying the willingness-to-pay threshold*: At a WTP of $887 (0.5x GDP *per capita*), two districts would not be allocated POC machines, and projected results would be 50.8% 30-day result-return, 48.2% 30-day ART initiation, 67.87 years (26.82 for CWH) undiscounted LE, costing $209/infant (discounted). At all WTP increases at or above $1,164, all 17 machines would be placed in the same locations as in the base-case analysis. As WTP increases further, health outcomes would remain unchanged, but NHB would increase as expected (equation (1)).

## Discussion

We employed a data-driven modeling approach to optimize health outcomes under resource constraints for EID programs in Matabeleland South, Zimbabwe, with four key findings. First, we demonstrate the feasibility of linking a validated microsimulation model and a novel optimization model. The primary aim of this linked model framework is to strategically locate POC machines in healthcare facilities with a focus on maximizing long-term clinical outcomes for infants and economic value for HIV programs. This approach combines the detailed disease progression dynamics captured by the CEPAC-Pediatrics model with the mathematical capabilities of the location-optimization model. This framework comprehensively evaluates the health and economic outcomes of EID testing approaches, including the use of both POC devices and conventional laboratory testing methods.

Second, optimally locating both existing and new POC machines markedly increases projected undiscounted life expectancy for CWH. For example, optimally locating the 17 currently available machines would increase projected LE among CWH from 25.91 years to 27.04 years (+1.13 life-years), reflecting the substantial impact of early ART initiation. The impact on LE among the entire modeled cohort is much smaller (increasing from 67.85 years with current location to 67.88 years with optimal location, or +0.4 life-months); this occurs because LE gains accrue only to the subset of CWH. Importantly, the full-cohort (including children with and without HIV) LE gain projected in this analysis is consistent with other modeled LE gains at the general population level for HIV testing in adults (+1.0 months), as well as other key healthcare interventions such as mammography (+0.8 months) or newborn Hepatitis B vaccination (+0.26 months) [42,44–46].

Third, decision tools based on modeling prove particularly valuable when making decisions regarding newly available resources amid constraints. This linked model can inform placement of newly acquired POC machines, with or without moving existing machines, to fit a range of goals, including maximizing clinical benefit or economic value while ensuring geographic equity. Moving machines from current locations may be operationally infeasible, costly, politically unpopular, or unethical because it removes access to existing services. This analysis did not account for these potential impacts. If the financial cost of moving machines were included, we anticipate that our modeled scenario of adding new machines to the existing program would become even more cost-effective.

We evaluated the advantages and disadvantages of various POC placement strategies, considering allocative efficiencies/location optimization, as well as pragmatic health system considerations. Prioritizing clinical benefit led to health gains for CWH, but required relocating devices from their current positions, which may be infeasible and unpopular. Adding machines sequentially demonstrated expected incremental improvements in health outcomes, although the returns diminished with each additional device. Importantly, maximizing NHB produced the same optimal allocation and projected outcomes as maximizing LE under the current WTP threshold, based on Zimbabwe’s GDP *per capita*. Lastly, the geographic equity scenario aimed to improve access by ensuring that each district had at least one machine. This required only one additional device, resulting in modestly higher cost and achieving nearly identical health outcomes compared to the base-case optimal placement strategy. Alternative equity objectives may also be relevant in settings where policymakers wish to address disparities more comprehensively. Such objectives could include allocating devices by targeting remote or hard‑to‑reach areas; or explicitly reducing between‑district disparities in outcomes such as 30‑day result‑return or ART initiation. While these approaches were beyond the scope of the current analysis, our modeling framework can accommodate them, and they represent important opportunities for future work to explore a broader range of equity/efficiency tradeoffs.

Our extensive deterministic sensitivity analyses demonstrate that the optimal allocations of the 17 devices remained identical to the base case, with the exception of extreme variations in POC test costs (doubling or halving). This stability demonstrates that our allocation recommendations are robust to uncertainty in model inputs and are not driven by narrow assumptions.

Finally, the choice of WTP threshold has a significant impact on the estimated value of POC infant HIV testing. The cost-effectiveness of HIV testing programs – for adults as well as children – is in large part driven by the long-term costs of caring for PLWH; although testing and treatment markedly increase LE, they also increase costs, and the choice of cost-effectiveness threshold greatly impacts whether testing programs are considered cost-effective [28,47,48]. As WTP increased, machines were added preferentially at sites where high test positivity rates improved the value of POC testing. To allocate POC machines in a way that maximized LE, a program must be willing to pay at least $1,164/YLS, at which point POC testing is considered cost-effective in all facilities. Our study demonstrates the sensitivity of optimal allocation to the available resources, as well as the importance of aligning POC device allocation with the broader healthcare objectives, while also considering financial realities of the healthcare system. Importantly, when maximizing LE, our model rendered identical optimal allocations as when maximizing NHB. In this setting, both LE and NHB are driven primarily by improved early ART initiation among CWH, while differences in testing costs between the *POC* and *LAB* strategies are relatively small compared to lifetime HIV care costs. Consequently, once the WTP threshold is high enough that the health gains of early diagnosis outweigh testing cost differences across facilities (≥$1,164/YLS), the allocation that maximizes LE also maximizes NHB. This aligns with previous work, which showed that EID is cost-effective, with impact maximized with high result-return [28], and that pediatric ART is cost-effective [40].

This analysis, conducted by a multidisciplinary team including program implementers and members of the Zimbabwean Ministry of Health and Child Care, provides valuable information for prioritizing limited resources in a high HIV prevalence setting. In the setting of increasingly scarce funding and resources, tools such as this one, which aim to maximize clinical outcomes and efficiencies, are critical. These tools can only be valuable if made widely available to program implementers. Additionally, beyond the scope of our modeling, additional programmatic factors will also be critical for improving diagnosis and survival. These include strengthening result-return systems, reducing delays in linkage to care, and ensuring consistent testing supply availability.

Our study has several limitations. First, data about assay costs and result-return probability likely reflect both under- and over-estimates in some sites. For example, we excluded the costs of moving existing POC machines to new locations due to lack of data, and some sites at which we modeled laboratory testing may in fact have sent samples to neighboring facilities with a POC machine under a “hub-and-spoke” model (underestimating both costs and result-return rate); conversely, the costs for all tests including those using existing POC devices were modeled as amortized total cost per test conducted, not as “sunk costs” (potentially overestimating costs for machines that were moved rather than added) [22]. We also used Global Fund data to inform laboratory-based testing costs in lieu of Zimbabwe-specific data. We evaluated the impact of these uncertain costs and result-return probabilities through extensive sensitivity analyses. Second, our analysis did not explicitly model inter-facility specimen referral patterns in which facilities without POC machines may send samples to neighboring facilities with existing POC capacity. Fully incorporating hub-and-spoke dynamics would require detailed data on current referral networks, inter-facility transport costs and times, and capacity constraints at potential hub facilities—data not available at the time of this analysis. Despite this limitation, our optimal allocation likely identifies facilities that would naturally serve as hubs. Additionally, decentralized POC testing has been shown to improve 30-day result-return rates compared to centralized laboratory approaches, particularly when specimen transport is delayed [49,50]. The choice between decentralized versus hub-and-spoke placement depends on local infrastructure and current referral patterns, factors that warrant prospective evaluation as part of future implementation science work. Third, our analysis incorporated health facility-specific data for demand, but data on result-return rate and timing, and test positivity rate were only available at the district level during our period of interest. Outcomes were nonetheless calculated at the facility level by combining facility-specific demand with district-level estimates of these parameters. While this approach allowed us to reflect variations in demand across facilities, the use of district-level input may have reduced our ability to capture important differences in service delivery or HIV prevalence between individual facilities. Going forward, facility-level data could better inform POC device location, and recent advancements in integrated sample transportation systems and expanded use of laboratory information management systems in Zimbabwe will enable more granular data to inform future decision-making. Fourth, we assumed that adding POC machines to a clinic would not increase the number of infants presenting for testing at that site (impacting demand). We made this assumption for two reasons: first, although in theory the availability of a POC machine might be appealing to families and might increase presentation for testing, this has never been formally evaluated or demonstrated in available studies or programmatic data. Second, we utilized programmatic health-facility-based demand to inform these analyses. We only had data for families presenting for testing – and therefore could not accurately gauge the number of families who would have otherwise undergone testing if the nearest facility were assigned a POC machine. We also lack data on individual family residence location and therefore could not assess additional facilities to which a family might travel if those facilities were assigned POC machines. Further, we assumed that adding POC machines within a given clinic would not change patient factors for vertical transmission of HIV (impacting test positivity rate). Future work could incorporate population density, geographic data, and prevalence mapping to achieve a more targeted and equitable location of POC machines, ultimately improving healthcare access for those in areas most affected by the HIV epidemic.

It is important to note that optimization frameworks prioritize efficiency and not equity, unless specific equity-focused policies (“constraints”) or outcomes (“objectives”) are included. For example, while our model projected that locating devices in clinics that demonstrate higher POC machine functionality would maximize overall life expectancy and net health benefit, clinics with lower POC machine functionality likely have fewer resources or face specific operational challenges that reduce functionality. It may be beneficial for programs to allocate additional resources to these facilities, rather than restricting their access to POC technology. Our geographic equity scenario illustrates this key finding. Distributing one POC machine to each district improved geographic equity and resulted in slight health outcome gains. With 18 machines, the projected LE for CWH remained largely unchanged from the base case, while program costs increased slightly. This demonstrates that implementing equity-focused constraints can expand access without harming health outcomes, though it does require additional resources. Where we were able to introduce a specific equity-focused constraint, we found only minimal reduction in 30-day ART initiation and pediatric life expectancy if program planners prioritized geographic equity (at least one POC machine per district) [9]. Incorporating equity constraints and objectives into model-based optimization is a critical area for future research [51].

## Conclusions

Our study highlights the potential of data-driven modeling tools to inform efficient resource allocation as well as to empower policymakers to make informed choices in healthcare delivery. Our research emphasizes the practical application of these tools and highlights the opportunity to integrate such tools into healthcare decision-making to maximize the impact of constrained resources on community health and well-being.

## Supporting information

S1 Fig Flowchart depicting the linkage between the CEPAC-Pediatric microsimulation model and the computer-based location-optimization model. (PDF)

S1 Appendix Table S1: Additional model input parameters for the CEPAC-Pediatric model. Table S2: Six total sub-cohorts, modeled within CEPAC-P. Table S3: Summary of scenarios evaluated in a location-optimization analysis of point-of-care infant HIV testing devices in Zimbabwe. Table S4: One-way sensitivity analysis when maximizing life expectancy. Table S5: One-way sensitivity analysis when maximizing net health benefit
(DOCX)
