# Supplementary material for: Optimizing the location of point-of-care infant HIV testing devices in Zimbabwe to maximize impact and value: A modeling analysis
Source: PLoS One. 2026 Aug 28;21(8):e0350921. doi: 10.1371/journal.pone.0350921 (PMC13524248; doi:10.1371/journal.pone.0350921)

Figure S1*:* Flowchart depicting the linkage between the CEPAC-Pediatric microsimulation model and the computer-based location-optimization model

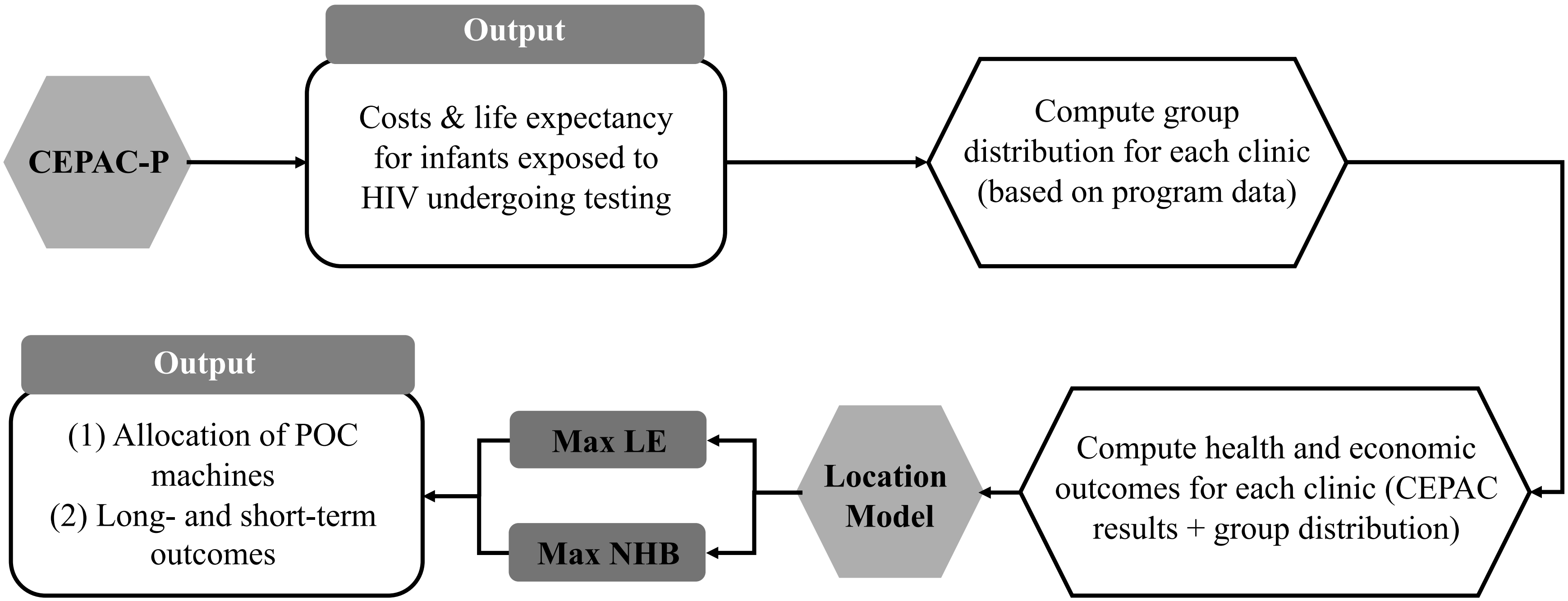

Supplement: Supplementary file 1 [file pone.0350921.s001.pdf]
