# Supplementary material for: Optimizing the location of point-of-care infant HIV testing devices in Zimbabwe to maximize impact and value: A modeling analysis
Source: PLoS One. 2026 Aug 28;21(8):e0350921. doi: 10.1371/journal.pone.0350921 (PMC13524248; doi:10.1371/journal.pone.0350921)
Supplement: Supplementary file 2 [file pone.0350921.s002.docx]

Supplementary online content for

**Optimizing the location of point-of-care infant HIV testing devices in Zimbabwe to maximize impact and value: A modeling analysis**

**Vivas-Valencia, *et al*.**

This supplementary material file has been created by the authors to give readers additional

information about the research article.

**Table of contents**

[1.	CEPAC-P model description and input parameters	3](#_Toc231199424)

[Table S1: Additional model input parameters for the CEPAC-Pediatric model	3](#_Toc231199425)

[2.	Integration of CEPAC-P and optimization model	5](#_Toc231199426)

[Step 1: Project long-term clinical and economic outcomes for all sub-cohorts within the CEPAC-Pediatric model	5](#_Toc231199427)

[Table S2: Six total sub-cohorts, modeled within CEPAC-P	6](#_Toc231199428)

[Step 2: Calculate the number of infants testing at each health facility, by strategy and testing modality	6](#_Toc231199429)

[Step 3: Calculate sub-cohort proportions, by strategy	7](#_Toc231199430)

[Step 4: Integration of the microsimulation and location-optimization models	9](#_Toc231199431)

[3.	Mathematical formulation of optimization model	10](#_Toc231199432)

[Parameters	10](#_Toc231199433)

[Mathematical formulation	11](#_Toc231199434)

[4.	Early infant diagnosis testing platforms	11](#_Toc231199435)

[Laboratory-based testing	11](#_Toc231199436)

[Point-of-care testing	12](#_Toc231199437)

[Confirmatory testing protocol	13](#_Toc231199438)

[5.	Additional calculations	13](#_Toc231199439)

[Calculating POC device functionality	13](#_Toc231199440)

[Assessing clinic need for multiple devices	14](#_Toc231199441)

[6.	Sensitivity analysis results	15](#_Toc231199442)

[Maximizing life expectancy (Table S4)	15](#_Toc231199443)

[Maximizing net health benefit (Table S5)	15](#_Toc231199444)

[Table S3: Summary of scenarios evaluated in a location-optimization analysis of point-of-care infant HIV testing devices in Zimbabwe	16](#_Toc231199445)

[Table S4: One-way sensitivity analysis when maximizing life expectancy	17](#_Toc231199446)

[Table S5: One-way sensitivity analysis when maximizing net health benefit	20](#_Toc231199447)

[References	23](#_Toc231199448)

# 1. CEPAC-P model description and input parameters

All infants simulated within CEPAC-P face monthly risks of non-HIV-related mortality, while infants with HIV face additional risks of opportunistic infections (OI) and mortality due to HIV-related causes. HIV can be diagnosed through routine infant testing programs offering user-specified diagnostic assays at various infant ages, or after development of an OI that prompts medical care. After HIV diagnosis, infants can link to HIV care and initiate ART; effective ART leads to reduced HIV RNA, increased CD4% (before age 5) or CD4 cell count (after age 5), and reductions in OI and mortality risk.

CEPAC-Pediatrics model inputs include CD4% at birth or at time of HIV acquisition; rates of CD4% decline, opportunistic infections, and mortality in the absence of antiretroviral therapy (ART); impact of ART on HIV RNA, CD4%, and opportunistic infection and mortality risk; and costs of HIV testing, clinical care, and ART (Tables 1 and S1). ART regimens and HIV-related costs were Zimbabwe-specific; where we lacked HIV disease progression data unique to Zimbabwe, we used inputs that were calibrated for Southern Africa.

| Table S1: Additional model input parameters for the CEPAC-Pediatric model | | |
| --- | --- | --- |
| **Parameter** | **Base-case value** | **Source** |
| **I. Input parameters used in CEPAC-Pediatric model** | | |
| **Infant cohort characteristics** | | |
| Proportion of mothers breastfeeding, % | 98.5 | ^1^ |
| Breastfeeding duration, mean (SD), months | 18 (6) | ^2^ |
| Proportion of infants receiving NVP as prophylaxis, % | 86 | ^3^ |
| Monthly risk of clinical events, by age, % |  |  |
| 0-5 years (range by age and CD4%) |  |  |
| WHO stage 3 event (except tuberculosis) | 3.3 – 11.6 | ^4^ |
| WHO stage 4 event (except tuberculosis) | 1.4 – 6.4 |  |
| Tuberculosis (any body site) | 0.5 – 3.8 |  |
| ≥ 5 years (range by CD4) |  |  |
| Mild fungal infection | 1.8 – 3.1 | ^5^ |
| Visceral bacterial infection | 0.04 – 0.71 |  |
| WHO stage 3 or 4 visceral disease | 0.03 – 1.43 |  |
| WHO stage 3 or 4 mucocutaneous disease | 0.03 – 2.26 |  |
| Other WHO stage 3 or 4 disease | 0.02 – 0.73 |  |
| Other severe disease | 0.19 – 1.67 |  |
| Other mild infection | 2.39 |  |
| Tuberculosis (any body site) | 0.03 – 1.74 |  |
| Risk of death within 30 days of clinical event, by age, % | | |
| 0 – 5 years |  |  |
| WHO Stage 3 or 4 event | 13.5 | ^4^ |
| Tuberculosis (any body site) | 11.1 |  |
| > 5 years |  |  |
| Visceral bacterial infection | 2.9 |  |
| WHO stage 3 or 4 visceral disease | 9.2 |  |
| WHO stage 3 or 4 mucocutaneous disease | 2.4 |  |
| Other WHO stage 3 or 4 disease | 20 |  |
| Other severe disease | 6.7 |  |
| Other mild infection | 0.4 |  |
| Tuberculosis (any body site) | 1.8 |  |

| **Parameter** | **Base-case value** | **Source** |
| --- | --- | --- |
| **Cost data, 2020 USD** |  |  |
| CD4 test | 4.06 | ^6^ |
| HIV viral load test | 24.05 | ^7^ |
| Monthly HIV care, by CD4 and ART status | 7.04 – 35.18 | ^8,9^ |
| Monthly pediatric ART (range by age and weight) |  |  |
| 1^st^-line | 5.65 – 11.70 | ^10,11^ |
| 2^nd^-line | 12.43 – 25.99 |  |
| 3^rd^-line | 30.68 – 46.57 |  |
| Monthly adult ART |  |  |
| 1^st^-line | 5.50 | ^6^ |
| 2^nd^-line | 23.00 |  |
| 3^rd^-line | 65.10 |  |
| ART, antiretroviral therapy; NVP, nevirapine; SD, standard deviation; USD, United States dollars; WHO: World Health Organization. | | |

# 2. Integration of CEPAC-P and optimization model

## Step 1: Project long-term clinical and economic outcomes for all sub-cohorts within the CEPAC-Pediatric model

Using the CEPAC-Pediatric model, we projected life expectancy and the associated costs for three groups of infants born to people living with HIV, including: (1) children without HIV (CWoH), (2) children with HIV (CWH) who do not receive test results, and (3) CWH who receive test results, assuming that 95% of infants who receive a positive HIV test result would initiate ART (as described in the main manuscript).^12^ (Importantly, result-return is not applied to children with negative results (CWoH), as costs and life expectancy remain unchanged irrespective of result-return status.) We ran each of these groups twice within the CEPAC-P model – once assuming all the children undergo laboratory-based testing, once assuming all undergo point-of-care (POC)-based testing. CEPAC-P then projected the life expectancy and lifetime care-related costs for each of these six model runs (3 groups of children x 2 strategies, Table S2).

| Table S2: Six total sub-cohorts, modeled within CEPAC-P | |
| --- | --- |
| CWoH[poc] | Children without HIV, undergoing POC-based testing |
| CWoH[lab] | Children without HIV, undergoing laboratory-based testing |
| CWH_RR[poc] | Children receiving +HIV test results after undergoing POC-based testing |
| CWH_RR[lab] | Children receiving +HIV test results after undergoing laboratory-based testing |
| CWH_nR[poc] | Children with +HIV POC-based test results who do NOT receive test results |
| CWH_nR[lab] | Children with +HIV laboratory-based test results who do NOT receive test results |

## Step 2: Calculate the number of infants testing at each health facility, by strategy and testing modality

Next, we determined how many children would present and undergo testing under both *POC* and conventional laboratory (*LAB*) strategies, using programmatic data for the number of infants seeking testing (demand, health facility-specific) for each health facility in Matabeleland South. For the *POC* strategy calculations, we also considered the district-specific POC device functionality (defined as the proportion of days when a POC machine is able to process testing samples), as well as the maximum throughput of an individual device (see *Assessing clinic need for multiple devices*, below). Consequently, if an infant arrives at a clinic where a POC machine is located but not functioning, or the device is functioning but the sample is collected after the device’s max daily throughput has been reached, the sample would be process via conventional laboratory approaches. The final outcomes computed for each health facility under the *POC* strategy thus reflect a mixed strategy of primarily POC testing, with some conventional laboratory testing being done when the POC machine is not functioning/has already met capacity. On the other hand, under the *LAB* strategy, all children who present to a health facility for testing undergo conventional laboratory-based testing.

We calculated results for each facility under both strategies in order to inform the optimization function (see Step 4).

Calculations: # of children undergoing testing at each facility, by strategy and testing modality:

- Under the *LAB* strategy, all children who present to a facility undergo conventional laboratory-testing

(1) #test[lab]_LAB = [demand(f)]

- Under the POC strategy, demand is split by district-facility functionality

(2) #test[poc]_POC = [demand(f)] x [functionality(d)]
(3) #test[lab]_POC = [demand(f)] x [1-functionality(d)]

*Note: (2) + (3) = (1)*

*“[lab]” indicates the type of testing an individual child undergoes
“_LAB” indicates a facility’s assigned strategy*

*(d) indicates a district-specific variable*

*(f) indicates a facility-specific variable*

## Step 3: Calculate sub-cohort proportions, by strategy

We next applied the test positivity rate (district-specific) and the testing modality-specific result-return rate for those with positive test results (applying 98% for POC-based testing^12,13^ and district-specific values for laboratory-based testing [Table 1]) to Step 2 calculations to determine the (1) the number of children who test positive for HIV within each facility and (2) the number of those children who receive their test results. This informs the proportions of the cohort who fall into the six sub-cohorts described in Step 1 for each strategy (*LAB* and *POC*). (Note: result-return is not applied to children whose test results are negative. Costs and life expectancy are expected to be the same for these children, regardless of whether they receive their test results or not.)

- For facilities assigned a POC device, the overall cohort will include all six sub-cohorts, including:

| #CWoH[poc] = | [1-test positivity(d)] x [#test[poc]_POC] |
| --- | --- |
| *The number of children with negative test results with POC-based testing.* *Result-return not applied, not impactful on outcomes.* | |
| #CWoH[lab] = | [1-test positivity(d)] x [#test[lab]_POC] |
| *The number of children with negative test results with laboratory-based testing. Result-return not applied, not impactful on outcomes.* | |
| #CWH_RR[poc] = | [test positivity(d)] x [+RR_test[poc]_POC]  Where [+RR_test[poc]_POC] = [#test[poc]_POC x 0.98] |
| *The number of children with returned positive test results with POC-based testing.* | |
| #CWH_RR[lab] = | [test positivity(d)] x [+RR_test[lab]_POC]  Where [+RR_test[lab]_POC] = [#test[lab]_POC x labRR(d)] labRR(d) = district-specific result-return for lab-based testing |
| *The number of children with returned positive test results with laboratory-based testing.* | |
| #CWH_nR[poc] = | [test positivity(d)] x [+nR_test[poc]_POC]  Where [+nR_test[poc]_POC] = [#test[poc]_POC x 0.02] |
| *The number of children with unreturned positive test results with POC-based testing.* | |
| #CWH_nR[lab] = | [test positivity(d)] x [+nR_test[lab]_POC]  Where [+nR_test[lab]_POC] = {#test[lab]_POC x [1-labRR(d)]} labRR(d) = district-specific result-return for lab-based testing |
| *The number of children with unreturned positive test results with laboratory-based testing.* | |

- If a facility is not assigned a POC device, then the overall cohort results include three sub-cohorts:

| #CWoH[lab] = | [1-test positivity(d)] x [#test[lab]_LAB] |
| --- | --- |
| *The number of children with negative test results with laboratory-based testing. Result-return not applied, not impactful on outcomes.* | |
| #CWH_RR[lab] = | [test positivity(d)] x [+RR_test[lab]_LAB]  Where [+RR_test[lab]_LAB] = [#test[lab]_LAB x labRR(d)] labRR(d) = district-specific result-return for lab-based testing |
| *The number of children with returned positive test results with laboratory-based testing.* | |
| #CWH_nR[lab] = | [test positivity(d)] x [+nR_test[lab]_LAB]  Where [+nR_test[lab]_LAB] = {#test[lab]_LAB x [1-labRR(d)]} labRR(d) = district-specific result-return for lab-based testing |
| *The number of children with unreturned positive test results with laboratory-based testing.* | |

## Step 4: Integration of the microsimulation and location-optimization models

Finally, the optimization model uses CEPAC-Pediatric output – the life expectancy and costs for each of the six sub-cohorts (Step 1) – together with the calculated number of children in each sub-cohort, by strategy (Step 3), to project outcomes for CWH and for the entire tested cohort at each facility, under both *LAB* and *POC* strategies (Figure S1). For each facility, we first pre‑compute the facility‑level totals for clinical and economic outcomes under both *POC* and *LAB* strategies by weighting CEPAC‑P outputs with the facility’s infant sub‑cohorts (Step 3). This process produces 122‑element vectors – one entry per facility – for each outcome (infant life expectancy [LE] or net health benefit [NHB]) under *POC* and *LAB*. These values enter the optimization model as fixed linear coefficients. The solver then selects the binary decision variable to choose between the *POC* or *LAB* value for each facility, depending on which outcome (LE or NHB) is being maximized.

The model considers all possible allocations of the available POC machines and their resulting clinical and economic outcomes. The objective function is used to identify the distribution of available POC machines among healthcare facilities that maximizes either infant LE or NHB.

# 3. Mathematical formulation of optimization model

We consider *j* health facilities indexed by *j ∈ {1,2, …, 122}* and districts indexed by *i ∈ {1,2, ..., 7}*. Infants arrive for testing at health facility *j* from district 𝑖 in month *t ∈ {1, ..., 12}* at a demand of *d_ijt_*. A binary decision variable *z_j_* indicates that a POC machine is assigned to health facility *j (z_j_ = 1)* or health facility *j* offers only laboratory-based testing (*z_j_* = 0). Therefore, *z = {z_1_ , z_2_, …, z_122_}* is a POC device relocation vector.

### Parameters

*l_j_*: Life expectancy with *POC* in facility *j*

*m_j_*: Life expectancy with *LAB* in facility *j*

*r_j_*: per-person NHB with *POC* in facility *j*

*a_j_:* per-person NHB with *LAB* in facility *j*

d_ijt_: number of infants arriving for testing at health facility j in district i in month t

K: total number of POC machines available for allocation (here, K = 17)

J(i): set of health facilities belonging to district i

Mathematical formulation
We maximize life expectancy or net health benefits from POC and conventional laboratory testing. The constraint ensures that a user-specific number of POC machines (here, 17) are assigned to health facilities.

$$Z_{j}=\left\{ \begin{aligned} 1, if a POC machine is assigned to health facility j \\ 0, if a POC machine is not assigned to health facility j \end{aligned} \right.$$

1) *Maximization of life expectancy*

Max	$\sum_{j\epsilon I} l_{j}z_{j}+ \sum_{j\epsilon I} m_{j} (1-z_{j})$

s.t. $\sum z_{j}=\boldsymbol{K}$

$\boldsymbol{z}_{\boldsymbol{j}}\boldsymbol{\in}\left\{ \boldsymbol{0,1} \right\}\boldsymbol{\forall}_{\boldsymbol{j}}$

2) *Maximization of net health benefit*

Max	$\sum_{j\epsilon I} r_{j}z_{j}+ \sum_{j\epsilon I} a_{j} (1-z_{j})$

s.t. $\sum z_{j}=\boldsymbol{K}$

$\boldsymbol{z}_{\boldsymbol{j}}\boldsymbol{\in}\left\{ \boldsymbol{0,1} \right\}\boldsymbol{\forall}_{\boldsymbol{j}}$

*Equity scenario:* In this scenario, geographic equity was enforced by adding the constraint

$\sum_{j\in\mathcal{J(}i)} z_{j}\geq1 \forall\text{ }i$,

requiring that each district $i$receive at least one POC device. This constraint was applied only in the equity scenario. All other aspects of the optimization model, including the linear objective and the binary decision variables, remained unchanged.

# 4. Early infant diagnosis testing platforms

## Laboratory-based testing

Conventional laboratory-based testing for early infant diagnosis within the Matabeleland South province is conducted using the Roche TaqMan Cobas 6800, which employs a qualitative nucleic acid test to detect HIV-1 DNA and RNA in dried blood spot samples from infants.^14^

## Point-of-care testing

The currently located 17 POC devices within the Matabeleland Province are a mix of GeneXpert and m-PIMA devices.

The GeneXpert instruments are supplied and serviced by Cepheid. These devices use the Xpert HIV-1 Qual assay for the qualitative in vitro diagnostic test to detect HIV Type 1 (HIV-1) target nucleic acids using real-time reverse transcription polymerase chain reaction (RT-PCR).^15^

m-PIMA analyzers are supplied by Abbott Rapid Diagnostics and serviced by Medsure. These devices employ the m-PIMA HIV 1/2 test, a quantitative in vitro nucleic acid amplification test designed to quantify HIV-1 groups M/N and O, and HIV type 2 RNA in human plasma specimens.^16^

Both GeneXpert and m-PIMA instruments have received WHO pre-qualification.^17^ These specific devices also underwent rigorous equipment validation and verification by the National Microbiology Reference Lab prior to deployment to facilities. All machines are operated by trained staff. The m-PIMA machines are operated by clinic staff (nurses and primary care counsellors). GeneXpert machines are operated by technical laboratory staff (laboratory scientists, laboratory technicians, and microscopists).

## Confirmatory testing protocol

Simulated infants undergo initial testing using either POC devices (POC strategy) or conventional laboratory-based testing (LAB strategy). For both strategies, positive test results are confirmed with POC polymerase chain reaction tests, conducted on new samples (i.e., separate from the sample on which the initial test was performed) drawn prior to ART initiation. If the second test is negative, ART is stopped.

# 5. Additional calculations

## Calculating POC device functionality

The Zimbabwe-based investigator team calculated POC device functionality based on a detailed analysis using information provided by facilities regarding equipment functionality and reagent availability reported in weekly T95 reports. The T95 is a routine report used to track the country’s progress towards the UNAIDS 95/95/95 targets, for which POC machines have been integral.

A functionality term was generated for each of the 17 available devices based on the information reported in the T95 reports. If a specific clinic did not have a POC device within the current allocation, we could not generate a clinic-specific functionality to apply if that clinic were allocated a device within a given scenario. Thus, instead of clinic-specific functionality, we calculated district-specific functionality based on the observed functionality of all POC devices allocated within a given district under the current scenario. If a district had one POC device, the functionality of that one device was applied as the functionality for that district for all simulated allocations. If a district had multiple POC devices in the current allocation, we averaged the functionality of all of those devices to calculate the district-specific functionality.

In the location-optimization model, we therefore simulated device functionality as the proportion of samples that are able to be successfully processed within a given week.

## Assessing clinic need for multiple devices

We needed to assess whether an individual clinic may have need for more than one POC device. In order to do this, we first calculated the yearly maximum throughput of a given POC device, based on time needed to execute a single test and staff availability to collect and process samples. We estimated this maximum throughput to be 1,300 tests per year.

We next applied the district-specific functionality (see above) – or the proportion of samples that are able to be successfully processed within a given week – to the max yearly throughput (assuming constant functionality throughout the year). This gave us a clinic-specific functional capacity.

Finally, we compared a doubling of the current clinic-specific yearly demand (reported in programmatic data) to the clinic-specific functional capacity. Doubling was chosen to reflect the extreme upper bound of the degree to which moving a POC device to a new clinic could potentially draw additional test-seeking families to that clinic, thus increasing the clinic-specific demand. In no cases did the doubled clinic-specific demand exceed the clinic-specific functional capacity. We thus concluded that one single POC device would be sufficient to meet the testing demand at all clinics.

# 6. Sensitivity analysis results

We conducted extensive deterministic sensitivity analyses, systematically varying key parameters (including monthly risk of loss to follow-up, risk of breastfeeding transmissions to infants who did not acquire HIV in pregnancy/delivery, and key cost parameters, including the cost of ART, HIV care, and POC tests) across their plausible ranges. The optimal device allocation remained constant across all these scenarios, demonstrating the robustness of our allocation recommendations to variation in model inputs.

### Maximizing life expectancy (Table S4)

With variation in key CEPAC-P parameters we found anticipated differences in projected LE and lifetime costs. However, the optimal location of the 17 currently available machines when optimizing LE remained the same as in the base-case scenario.

### Maximizing net health benefit (Table S5)

Varying monthly loss to follow-up, breastfeeding HIV acquisition risks and costs of ART and HIV-related care led to anticipated changes in projected LE and lifetime costs and thus calculated NHB. However, compared to the base case, the locations of the POC machines to maximize NHB at a WTP of $1,774 changed only with variation in the cost of POC tests.

| Table S3: Summary of scenarios evaluated in a location-optimization analysis of point-of-care infant HIV testing devices in Zimbabwe | | |
| --- | --- | --- |
| **Scenario** | **Description** | **Optimization goal** |
| Current placement (baseline) | 17 POC machines in 11 facilities, reflecting actual programmatic distribution as of 2023, mapped into 2019-2020 data | Descriptive  (not optimized) |
| Base case  (optimal placement) | Reallocation of the 17 POC machines available across facilities | Maximize LE or NHB |
| Adding POC machines to the current program | Begin with 17 current POC machines, add new machines one by one | Maximize LE |
| Geographic equity scenario | Begin with optimal placement of 17 devices, then add devices one by one with constraint that each district receives ≥1 device | Maximize LE |
| Varying WTP threshold | Evaluate NHB under WTP thresholds | Maximize NHB |
| LE, life expectancy; NHB, net health benefit; POC, point-of-care; WTP, willingness-to-pay. | | |

## Table S4: One-way sensitivity analysis when maximizing life expectancy

| **Outcomes** | **Base case** | **Care costs  0.5x** | **Care costs  2x** | **ART costs 0.5x** | **ART costs  2x** | **LTFU  0.5x** | **LTFU  2x** |
| --- | --- | --- | --- | --- | --- | --- | --- |
| Result-return, % | 52.62 | NC | NC | NC | NC | NC | NC |
| ART initiation, % | 50.00 | NC | NC | NC | NC | NC | NC |
| Cost, disc., USD (full cohort) | 211 | 164 | 330 | 185 | 264 | 235 | 178 |
| Cost, disc., USD (CWH) | 7,143 | 5,180 | 12,053 | 6,058 | 9,316 | 8,104 | 5,762 |
| Net health benefit, disc. (full cohort) | 27.75 | 27.77 | 27.68 | 27.76 | 27.72 | NC | 27.74 |
| Life expectancy, undisc. (full cohort) | 67.88 | NC | NC | NC | NC | 67.94 | 67.79 |
| Life expectancy, undisc. (CWH) | 27.04 | NC | NC | NC | NC | 29.32 | 23.17 |

| **Outcomes** | **Base case** | **Point-of-care testing input parameter** | | | | | | | |
| --- | --- | --- | --- | --- | --- | --- | --- | --- | --- |
|  |  | **Sensitivity 70%**** | **Sensitivity 80%**** | **Sensitivity 100%**** |  | **Specificity 95%**** | **Specificity 100%**** | **Costs 0.5x** | **Costs  2x** |
|  |  | **Base case = 96.9%** | | |  | **Base case = 99.6%** | |  |  |
| Result-return, % | 52.62 | 52.68 | 52.68 | NC |  | NC | NC | NC | NC |
| ART initiation, % | 50.00 | 50.05 | 50.05 | NC |  | NC | NC | NC | NC |
| Cost, discounted, USD (full cohort) | 211 | 205 | 208 | 212 |  | 212 | NC | 207 | 221 |
| Cost, discounted, USD (CWH) | 7,143 | 6,889 | 6,983 | 7173 |  | NC | NC | 7,140 | 7,151 |
| Net health benefit (full cohort) | 27.75 | 27.74 | 27.74 | NC |  | NC | NC | NC | 27.74 |
| Life expectancy, undisc. (full cohort) | 67.88 | 67.86 | 67.87 | NC |  | NC | NC | NC | NC |
| Life expectancy, undisc. (CWH) | 27.04 | 26.20 | 26.51 | 27.14 |  | NC | NC | NC | NC |

| **Outcomes** | **Base case** | **Laboratory-based testing input parameter** | | | | | | | | |  |
| --- | --- | --- | --- | --- | --- | --- | --- | --- | --- | --- | --- |
|  |  | **Sensitivity 70%**** | | **Sensitivity 80%**** | **Sensitivity 100%**** | |  | **Specificity 95%**** | **Costs 0.5x**** | **Costs  2x** |  |
|  |  |  | **Base case = 99.4%** | | |  |  | **Base case = 100%** |  |  |  |
| Result-return, % | 52.62 | NC | | NC | NC | |  | NC | NC | NC |  |
| ART initiation, % | 50.00 | NC | | NC | NC | |  | NC | NC | NC |  |
| Cost, discounted, USD (full cohort) | 211 | 207 | | 209 | NC | |  | 213 | 203 | 230 |  |
| Cost, discounted, USD (CWH) | 7,143 | 6,980 | | 7,035 | 7,147 | |  | NC | 7,136 | 7,158 |  |
| Net health benefit (full cohort) | 27.75 | 27.74 | | 27.74 | NC | |  | NC | NC | 27.74 |  |
| Life expectancy, undisc. (full cohort) | 67.88 | 67.87 | | 67.87 | NC | |  | 67.89 | NC | NC |  |
| Life expectancy, undisc. (CWH) | 27.04 | 26.51 | | 26.69 | 27.05 | |  | NC | NC | NC |  |
| ART, antiretroviral therapy; CWH, children with HIV; LTFU, loss to follow-up after initiation of HIV care; NC, no change; undisc, undiscounted; USD, 2020 United States dollars.  Discounted results are discounted at 3%/year. Costs are reported per person.  Blue indicates a decrease from the base-case value; orange indicates an increase from the base-case value.  ** Indicates sensitivity analysis in which the allocation of the 17 currently available devices changed compared to the base-case allocation. | | | | | | | | | | | |

## Table S5: One-way sensitivity analysis when maximizing net health benefit

| **Outcomes** | **Base case** | **Care costs 0.5x** | **Care costs  2x** | **ART costs  0.5x** | **ART costs  2x** | **LTFU  0.5x** | **LTFU  2x** |
| --- | --- | --- | --- | --- | --- | --- | --- |
| Result-return, % | 52.62 | 52.68 | NC | NC | NC | NC | NC |
| ART initiation, % | 50.00 | 50.05 | NC | NC | NC | NC | NC |
| Cost, discounted, USD (full cohort) | 211 | 164 | 330 | 185 | 264 | 235 | 178 |
| Cost, discounted, USD (CWH) | 7,143 | 5,176 | 12,053 | 6,058 | 9,316 | 8,104 | 5,762 |
| Net health benefit (full cohort) | 27.75 | 27.77 | 27.68 | 27.76 | 27.72 | NC | 27.74 |
| Life expectancy, undisc. (full cohort) | 67.88 | NC | NC | NC | NC | 67.94 | 67.79 |
| Life expectancy, undisc. (CWH) | 27.04 | NC | NC | NC | NC | 29.32 | 23.17 |

| **Outcomes** | **Base case** | **Point-of-care testing input parameter** | | | | | | | |
| --- | --- | --- | --- | --- | --- | --- | --- | --- | --- |
|  |  | **Sensitivity 70%** | **Sensitivity 80%** | **Sensitivity 100%** |  | **Specificity 95%** | **Specificity 100%** | **Costs 0.5x**** | **Costs  2x**** |
|  |  | **Base case = 96.9%** | | |  | **Base case = 99.6%** | |  |  |
| Result-return, % | 52.62 | NC | NC | NC |  | NC | NC | 52.68 | 50.75 |
| ART initiation, % | 50.00 | NC | NC | NC |  | NC | NC | 50.05 | 48.21 |
| Cost, discounted, USD (full cohort) | 211 | 205 | 208 | 212 |  | 212 | 211 | 206 | 215 |
| Cost, discounted, USD (CWH) | 7,143 | 6,888 | 6,982 | 7,173 |  | NC | NC | 7,139 | 7,085 |
| Net health benefit (full cohort) | 27.75 | 27.74 | 27.74 | NC |  | NC | NC | NC | 27.74 |
| Life expectancy, undisc. (full cohort) | 67.88 | 67.86 | 67.87 | NC |  | NC | NC | NC | 67.87 |
| Life expectancy, undisc. (CWH) | 27.04 | 26.20 | 26.51 | 27.14 |  | NC | NC | NC | 26.82 |

| **Outcomes** | **Base case** | **Laboratory-based testing input parameter** | | | | | | | | | |
| --- | --- | --- | --- | --- | --- | --- | --- | --- | --- | --- | --- |
|  |  | **Sensitivity 70%** | | **Sensitivity 80%** | **Sensitivity 100%** | |  | **Specificity 95%** | **Costs 0.5x** | **Costs  2x** | |
|  |  |  | **Base case = 99.4%** | | |  |  | **Base case = 100%** |  |  |  |
| Result-return, % | 52.62 | NC | | NC | NC | |  | NC | 52.28 | 52.68 | |
| ART initiation, % | 50.00 | NC | | NC | NC | |  | NC | 49.67 | 50.05 | |
| Cost, discounted, USD (full cohort) | 211 | 207 | | 209 | NC | |  | 213 | 202 | 230 | |
| Cost, discounted, USD (CWH) | 7,143 | 6,980 | | 7,035 | 7,147 | |  | NC | 7,125 | 7,158 | |
| Net health benefit (full cohort) | 27.75 | 27.74 | | 27.74 | NC | |  | NC | NC | 27.74 | |
| Life expectancy, undisc. (full cohort) | 67.88 | 67.87 | | 67.87 | NC | |  | 67.89 | NC | NC | |
| Life expectancy, undisc. (CWH) | 27.04 | 26.51 | | 26.69 | 27.05 | |  | NC | 27.00 | NC | |
| ART, antiretroviral therapy; CWH, children with HIV; LAB, laboratory-based testing for early infant HIV diagnosis; LTFU, loss to follow-up after initiation of HIV care; NC, no change; POC: point-of-care testing for early infant HIV diagnosis. undisc, undiscounted; USD, 2020 United States dollars.  Discounted results are discounted at 3%/year. Costs are reported per person.  Blue indicates a decrease from the base-case value; orange indicates an increase from the base-case value.  ** Indicates sensitivity analysis in which the allocation of the 17 currently available devices changed compared to the base-case allocation. | | | | | | | | | | |  |

# References

1.	ZIMPHIA. Zimbabwe population-based HIV impact assessment ZIMPHIA 2020 key findings. 2020. Accessed September 26, 2022. https://phia.icap.columbia.edu/wp-content/uploads/2020/11/ZIMPHIA-2020-Summary-Sheet_Web.pdf

2.	Zimbabwe National Statistics Agency (ZIMSTAT), UNICEF. *Zimbabwe Multiple Indicator Cluster Survey 2019, Survey Findings Report*. ZIMSTAT and UNICEF; 2019. Accessed August 4, 2020. https://mics-surveys-prod.s3.amazonaws.com/MICS6/Eastern%20and%20Southern%20Africa/Zimbabwe/2019/Survey%20findings/Zimbabwe%202019%20MICS%20Survey%20Findings%20Report-31012020_English.pdf

3.	Desmond AC, Moodley D, Conolly CA, Castel SA, Coovadia HM. Evaluation of adherence measures of antiretroviral prophylaxis in HIV exposed infants in the first 6 weeks of life. *BMC Pediatr*. 2015;15:23. doi:10.1186/s12887-015-0340-9

4.	Ciaranello A, Lu Z, Ayaya S, et al. Incidence of World Health Organization stage 3 and 4 events, tuberculosis and mortality in untreated, HIV-infected children enrolling in care before 1 year of age: an IeDEA (International Epidemiologic Databases To Evaluate AIDS) East Africa regional analysis. *Pediatr Infect Dis J*. 2014;33(6):623-629. doi:10.1097/INF.0000000000000223

5.	Holmes CB, Wood R, Badri M, et al. CD4 decline and incidence of opportunistic infections in Cape Town, South Africa: implications for prophylaxis and treatment. *J Acquir Immune Defic Syndr*. 2006;42(4):464-469. doi:10.1097/01.qai.0000225729.79610.b7

6.	The Global Fund. *Pooled Procurement Mechanism Reference Pricing: ARVs (Version: Quarter 3 2020)*. The Global Fund; 2020. https://www.theglobalfund.org/media/5813/ppm_arvreferencepricing_table_en.pdf

7.	The Global Fund. Viral Load & Early Infant Diagnosis | HIV Viral Load and Early Infant Diagnosis Selection and Procurement Information Tool. April 2017. Accessed November 8, 2024. https://www.theglobalfund.org/en/sourcing-management/health-products/viral-load-early-infant-diagnosis/

8.	Menzies NA, Berruti AA, Berzon R, et al. The cost of providing comprehensive HIV treatment in PEPFAR-supported programs. *AIDS*. 2011;25:1753-1760. doi:10.1097/QAD.0b013e3283463eec

9.	Magure T, Manenji A, Gboun M, et al. *Zimbabwe National AIDS Spending Assessment: Consolidated Report 2011 and 2012*. National AIDS Council of Zimbabwe and UNAIDS; 2012.

10.	The Global Fund. Price & Quality Reporting Price Reference Report. Tableau Software. 2020. Accessed February 22, 2022. https://public.tableau.com/views/PQRPricelist_English/PriceList?%3Adisplay_static_image=y&%3AbootstrapWhenNotified=true&%3Aembed=true&%3Alanguage=en-US&:embed=y&:showVizHome=n&:apiID=host0#navType=0&navSrc=Parse

11.	Doherty K, Essajee S, Penazzato M, Holmes C, Resch S, Ciaranello A. Estimating age-based antiretroviral therapy costs for HIV-infected children in resource-limited settings based on World Health Organization weight-based dosing recommendations. *BMC Health Serv Res*. 2014;14:201. doi:10.1186/1472-6963-14-201

12.	Bianchi F, Cohn J, Sacks E, et al. Evaluation of a routine point-of-care intervention for early infant diagnosis of HIV: an observational study in eight African countries. *Lancet HIV*. 2019;6(6):e373-e381. doi:doi:%2010.1016/S2352-3018(19)30033-5.

13.	Elizabeth Glaser Pediatric AIDS Foundation. Point-of-Care Early Infant Diagnosis Data Dashboard. June 20, 2019. Accessed October 6, 2022. https://www.pedaids.org/impact/data-dashboard/point-care-early-infant-diagnosis-data-dashboard/

14.	*Cobas® 6800 System*. Roche Diagnostics. Accessed October 20, 2025. https://diagnostics.roche.com/us/en/products/instruments/cobas-6800-ins-2693.html

15.	*Cepheid GeneXpert® Systems*. Cepheid. Accessed October 20, 2025. https://www.cepheid.com/en-US/systems/genexpert-family-of-systems.html?utm_medium=cpc&utm_source=google&utm_campaign=2025-NAM-US-EN-CHILD-PPC-SEARCH-BRAND-PCRPlus&utm_term=%7Bkeyword%7D&LeadCampaignID=701Nu00000H39eTIAR

16.	*M-PIMA^TM^ HIV-1/2 Detect*. Abbott. Accessed October 20, 2025. https://www.globalpointofcare.abbott/ww/en/product-details/m-pima-hiv-12-detect.html

17.	World Health Organization (WHO). *WHO List of Prequalified In Vitro Diagnostic Products*. 2025. Accessed October 27, 2025. https://extranet.who.int/prequal/vitro-diagnostics/prequalified/in-vitro-diagnostics?page=2
